# Comparative Evaluation of Transdermal Formulations of Norfloxacin With Silver Sulfadiazine Cream, USP, for Burn Wound Healing Property

**Published:** 2006-06-07

**Authors:** Venkata Ramana Malipeddi, Kamal Dua, Udai Vir Singh Sara, Himaja Malipeddi, Abhinav Agrawal

**Affiliations:** a NGSM Institute of Pharmaceutical Sciences, Mangalore, Karnataka; b D. J. College of Pharmacy, Modinagar, Uttar Pradesh, India

## Abstract

**Objective:** In an attempt to find a better treatment for bacterial infections and burn wounds, various semisolid formulations containing 5% w/w of norfloxacin were prepared and evaluated for physicochemical parameters, in vitro drug release through cellophane membrane, antimicrobial activity, and burn wound healing properties. The prepared formulations were compared with silver sulfadiazine 1% cream, USP. **Methods:** Various semisolid formulations were prepared with different bases like Carbopol, polyethylene glycol, and hydroxypropylmethyl cellulose, using standard procedures. The antimicrobial activity of these semisolid norfloxacin formulations, against various strains of aerobic and anaerobic microorganisms, was evaluated by using a standard cup-plate method. The wound healing property was evaluated by measuring the wound contraction and expressed as percentage of contraction of original wound size for each animal group. **Results:** Antimicrobial activity of norfloxacin semisolid formulations was found to be equally effective against both aerobic and anaerobic bacteria in comparison to a formulation of silver sulfadiazine 1% cream, USP, available on the market. **Conclusion:** The burn wound healing property of the prepared norfloxacin semisolid formulations was found to be in good agreement with silver sulfadiazine 1% cream, USP, available on the market.

Infection is a major complication of burn injury and is responsible for 50% to 75% of hospital deaths. A moist, thermally coagulated burn wound, with its constantly replenished supply of diffusing serum nutrients and warm surface temperature, provides an environment suitable for rapid microbial growth. As local microbial growth increases, the potential for invasion to subjacent viable tissues and penetration into circulation increases.

Microorganisms that cause burn wound infections have changed over the years relative to changes in treatment. Ramakrishanan et al^1^ and Wang et al^2^ reported separately that anaerobic bacteria are the causative organisms for infection in around 15% of burn-infected patients. Huo^3^ reported that silver norfloxacin proved valuable in the treatment of burn wound infection caused by invading organisms, particularly by a silver sulfadiazine--resistant strain of *Pseudomonas.*

Norfloxacin, a broad-spectrum fluoroquinolone antibacterial agent, is commonly employed in the treatment of urinary and genital tract infections.^4^–^6^ It is a hydrophilic fluoroquinolone with unique physiochemical properties such as low water solubility and partition coefficient.^7^ The objective of the present study was to prepare various topical drug delivery systems such as gels and ointments and evaluate their antimicrobial activity and burn wound healing efficacy and compare with silver sulfadiazine 1% cream, USP.

## MATERIALS AND METHODS

Norfloxacin was obtained from Pfiscar India Ltd. (Murthal, Haryana, India) and Carbopol from Noveon (Mumbai, India). All other chemicals used were of analytical grade. For analysis, UV-spectrophotometer (Jasco V-530; Jasco Inc, Easton, MD) was used.

### 

#### Preparation of transdermal formulations

Various semisolid formulations of norfloxacin were prepared, with different bases like Carbopol, polyethylene glycol, and hydroxypropylmethyl cellulose using standard procedures (Table 1). In each of the formulations, norfloxacin was incorporated at 5% w/w concentration in the base with trituration using geometric dilution procedure to get a homogeneous mass.

### Evaluation of transdermal formulations

#### Drug content

A hundred milligrams of formulation was dissolved in 1% v/v acetic acid, filtered, and the volume was made to 100 mL with 1% v/v acetic acid. The resultant solution was suitably diluted with 1% v/v acetic acid and absorbance was measured at 277.6 nm. From this the drug content was determined using calibration curve for norfloxacin.

#### pH

Direct measurements were made using a digital pH meter.

### Rheological studies

The prepared formulations were evaluated for the following rheological characteristics (Table 2):

- *Apparent viscosity* at 2.5 rpm, using a Brookfield synchroelectric viscometer.
- *Spreadability*, using a spreadability apparatus.^8^ After applying weight, time in seconds required to separate the slides was noted. Spreadability of each formulation was reported in seconds.
- *Extrudability*, using an extrudability apparatus.^9^ A closed collapsible tube containing formulation was pressed firmly at the crimped end. When the cap was removed, formulation extruded until the pressure dissipated. Weight in grams required to extrude a 0.5-cm ribbon of the formulation in 10 seconds was determined. The average extrusion pressure in grams was reported.

### In vitro release studies

In vitro release studies were carried out using the classical cylindrical tube (Table 3).^10^ Semisolid formulation (0.5 g) was taken on the cellophane membrane and tied securely to one end of the tube, the other end was kept open to ambient conditions. The cell was inverted and immersed slightly in 100.0 mL of 1% v/v acetic acid in a beaker at 37°C ± 1°C, and stirred at 100 rpm for 8 hours. Samples of 5.0 mL were withdrawn at 1-hour intervals and assayed spectrophotometrically at 277.6 nm.

### Microbiological studies

The antibacterial activity of various semisolid formulations of norfloxacin against various strains of aerobic and anaerobic microorganisms was evaluated by the standard cup-plate method and the inhibition zone diameters were measured with the help of a zone reader (Table 4). *Bacillus subtilis*, *Staphylococcus aureus*, *Escherichia coli*, *Pseudomonas aeruginosa* (aerobic organisms) and *Bacteroides fragilis* (anaerobic organism) were used for testing the antibacterial activity. Nutrient agar medium was used for aerobic bacterial cultures and blood agar medium was used for *Bacteroides fragilis*. The aerobic organisms were incubated at a temperature of 37°C ± 0.2°C for 24 hours in an incubator under aerobic conditions, while *Bacteroides fragilis* cultures were incubated under a hydrogen/carbon dioxide atmosphere in an anaerobic jar at 37°C ± 0.2°C for 48 hours.^11^

### Burn wound healing property

#### Animals

Healthy Wistar albino rats weighing between 150 and 180 g were used. Animals were divided into several groups, each containing 5 animals.

#### Inflicting burn wound

The experiments were carried out per the guidelines of Animal Ethics Committee. The dorsum of each rat was shaved. Burn wounds were inflicted on overnight-starved animals under pentobarbitone sodium (6 mg/100 g, i.p.) anesthesia. A 2 × 2-cm metal cylinder was placed on the shaven back of the animals. Melted wax at 80°C was poured into the metal cylinder and the wax was allowed to solidify. Eight minutes after this, until the wax was completely solidified, the metal cylinder containing wax adhering to the skin was gently removed to inflict a distinctly demarked burn wound.^12^

### Assessment of burn wound healing

Norfloxacin semisolid formulations and silver sulfadiazine 1% cream, USP, available on the market were applied (500 mg each) to the wound inflicted areas of animals every day from day 1. Animals were observed for wound healing by measuring the wound contraction (tracing the raw wound area first on a transparent polythene paper and then retraced on graph paper) up to the 12th day postwounding. The wound contraction was calculated as percentage of original wound size for each animal of a group (Table 5).^12^

## RESULTS AND DISCUSSION

The drug content of all the formulations was found to be in good agreement with the theoretical value, indicating the stability of the drug in the formulations. pH of all the formulations was found to be between 5.7 and 7.2, which indicated suitability of the formulations for application on the skin. Rheological properties (spreadability and extrudability) of the norfloxacin semisolid formulations were found to be equivalent to silver sulfadiazine cream USP.

Norfloxacin formulations containing polyethylene glycol and Carbopol gel base showed better in vitro release profile and gave larger zones of inhibition in comparison to creams and ointment base formulations, indicating the better activity of the drug. In creams, owing to their biphasic nature, partitioning of the drug occurs in 2 phases, resulting in slower release of drug, while in the case of gels, the drug diffusion occurs through the aqueous phase and hence they offer a greater drug diffusion and release.^13^

The difference between the mean percent burn wound contraction of the norfloxacin formulation treated animals as compared to control was found to be statistically significant. All the prepared formulations, including silver sulfadiazine 1% cream, USP, had statistically shown wound healing activity (*P* = .001) in healthy male Wistar albino rats. Burn wound healing studies revealed a maximum percent wound healing of 83.01% ± 0.73% with carbopol formulations within 12 days, which is in good agreement with the silver sulfadiazine formulation available on the market, showing 84.34% ± 0.49% of wound healing.

## CONCLUSION

The in vitro release characteristics along with the burn wound healing property of prepared topical formulations of Norfloxacin were quite encouraging and in good agreement with the silver sulfadiazine 1% cream, USP, available on the market. Among all the semisolid formulations prepared, Carbopol gel base was found to be most suitable dermatological base for norfloxacin in comparison with various other dermatological bases. It also has aesthetic appeal, which other bases lack, an important aspect from patient compliance and consumer point of view.

## Figures and Tables

**Table 1:** Composition of various optimized dermatological bases containing 5% w/w norfloxacin

	**Dermatological base**			
	
**Ingredients**	**Carbopol, g**	HPMC, g*	**PEG, g**	**Ointment, g**
Norfloxacin	5.0	5.0	5.0	5.0
Carbopol gel base	95.0	…	…	…
Propylene glycol	…	…	…	…
Methyl paraben	…	0.3	…	…
Ethyl paraben	…	0.2	…	10.0
HPMC gel base	…	2.0	…	…
PEG 4000	…	…	50.0	…
PEG 300	…	…	45.0	…
DMSO	…	…	…	10.5 v/w
Isopropyl myristate	…	…	…	8.0 v/w
Mineral oil	…	…	…	30.0 v/w
White petrolatum	…	…	…	30.7 v/w
Bees wax	…	…	…	4.8 v/w
Glycerine	…	10.0	…	…
Water	…	q.s.	…	1.0 v/w

*HPMC indicates hydroxypropylmethyl cellulose; PEG, polyethylene glycol; DMSO, dimethyl sulfoxide; and q.s., quantity sufficient.

**Table 2:** Physicochemical properties of semisolid formulations of norfloxacin*

**Formulation base**	**% Drug content**	**pH**	**Apparent viscosity, cPs**	**Flow index**	**Spreadability, s**	**Extudability, g**
Carbopol	97.4 ± 2.8	7.2	10 × 10^6^	0.35	25	500
PEG†	93.1 ± 3.7	6.3	30 × 10^6^	0.26	38	520
HPMC	99.7 ± 4.8	5.7	45 × 10^6^	0.37	45	578
Ointment	95.3 ± 4.7	6.1	25 × 10^6^	0.22	35	589

*Values are average of 3 determinations.

^†^PEG indicates polyethylene glycol; HPMC, hydroxypropylmethyl cellulose.

**Table 3:** In vitro drug release profile of semisolid formulations of norfloxacin

Formulation base	% Drug release after 8 h*
Carbopol	75.03 ± 3.31
PEG†	69.74 ± 2.28
HPMC	49.13 ± 2.79
Ointment	43.00 ± 3.79
Silver sulfadiazine 1% cream, USP	71.17 ± 3.33

*Values are average of 3 determinations.

^†^PEG indicates polyethylene glycol; HPMC, hydroxypropylmethyl cellulose.

**Table 4:** Antimicrobial activity of norfloxacin from topical drug delivery system

	Inhibition zone diameter, mm*				
	
**Formulation base**	***Bacillus subtilis***	***Staphylococcus aereus***	***Escherichia coli***	***Pseudomonas aeruginosa***	***Bacteroides fragilis***
Carbopol	48.82	50.82	47.32	45.33	49.33
PEG†	43.72	42.33	41.22	42.32	46.21
HPMC	36.32	33.33	31.21	32.33	35.21
Ointment	32.13	31.33	30.23	30.21	30.11
Silver sulfadiazine 1% cream, USP	45.33	51.22	48.31	44.72	47.12

*Values are average of 3 determinations.

^†^PEG indicates polyethylene glycol; HPMC, hydroxypropylmethyl cellulose.

**Table 5:** Percent burn wound contraction of norfloxacin formulations

	% Wound contraction		
	
**Formulation base**	**Day 4**	**Day 8**	**Day 12**
Control	27.61 ± 1.33*	53.31 ± 1.22*	67.03 ± 0.92*
Carbopol	49.13 ± 1.56*	70.00 ± 1.86*	83.01 ± 0.73*
Carbopol†	31.03 ± 1.84*	58.13 ± 0.37*	70.10 ± 0.92*
PEG	46.12 ± 1.72*	67.23 ± 0.32*	78.00 ± 0.67*
PEG†	30.02 ± 1.30v	56.34 ± 0.24*	65.31 ± 0.73*
HPMC	43.13 ± 1.36*	59.76 ± 1.21*	69.65 ± 0.87*
HPMC†	30.10 ± 0.72*	48.01 ± 0.29*	58.03 ± 0.29*
Ointment	40.23 ± 1.37*	57.00 ± 0.78*	62.65 ± 0.13*
Ointment†	29.01 ± 0.93*	57.13 ± 0.29*	61.00 ± 0.27*
Silver sulfadiazine 1% cream, USP	52.00 ± 1.45*	71.87 ± 0.98*	84.34 ± 0.49*

*P < .001 vs control.

^†^Represent the group treated with base alone.
